# Supplementary material for: Archaeometric analysis of Early Bronze Age bread from Küllüoba Höyük
Source: PLoS One. 2026 Mar 27;21(3):e0344705. doi: 10.1371/journal.pone.0344705 (PMC13028366; doi:10.1371/journal.pone.0344705)
Supplement: Supplementary file 1 [file pone.0344705.s001.docx]

Archaeobotanical Data from Küllüoba Höyük Trench T8																								
		**Sample No.**	**T8 32/1**	**T8 32/5**	**T8 34/2**	**T8 34/4**	**T8 35/1**	**T8 35/2**	**T8 35/3**	**T8 36/5**	**T8 36/11**	**T8 36/12**	**T8 37/1**	**T8 37/2**	**T8 38/1**	**T8 39/1**	**T8 44/1**	**T8 45/2**	**T8 45/3**	**T8 46/1**	**T8 46/3**	**T8 46/5**	**T8 46/6**	**T8 48/1**
		**Context**	Hearth	Inside the Pot	Infill	Pottery Side	In-Field South-East	In front of the hearth	In-Field North-East	Ceramic side	Inside the Amphora	Inside the Bowl	Pit (Upper Level)	Pit (Base Level)	In front of the hearth	Inside the Pit	Inside the Hearth	Hearth base	In front of the hearth	Hearth	East of the Hearth	Area Surface	Pit	Hearth
		**Period**																						
		**Volume (lt)**	2,8	2	3,4	3,2	3,1	3	3,5	2,7	0,35	1,5	3	3,2	3	3	2,5	5	3	2,8	2,5	3,2	2,7	3,4
**Taxon**	**English Name**	**Component**																						
**Agricultural Plants**																								
Hordeum vulgare	barley	grain		1	24	14	14	4	23	7	2		15	11	9	53	3	8	5	21	19	397	13	47
Hordeum vulgare	barley	spikelet fork		5	1	3	2	2		1			7	1		4		2		2	7	73		6
Triticum monococcum	einkorn wheat	grain		1	13	10	6		4	6			13	1	4	25	3		3	6	14	6	7	25
Triticum dicoccum	emmer wheat	grain		5	73	10	11	5	17	2	3	3	27	14	7	28	4		1	5	37	16	2	34
Triticum aestivum/durum	bread/durum wheat	grain			10	6	2	1	5	1			11	2	4	4	6	1		1	5	2	4	1
T. monococcum/dicoccum	einkorn/emmer wheat	spikelet fork		7	81	29	16	6	64	6	9	7	91	72	37	12	1	51	4	16	55		17	39
Triticum sp./*Hordeum* sp.	wheat/barley	grain fragments	4	5	107	23	16	9	102	18	9	8	27	18	26	9	10	24	14	26	36	87	23	32
Lathyrus sativus	grass pea	seed																	1				1	
Lens culinaris	lentil	seed	1	3	7	1	4			2				3	3	6		3	4	4	4	3	1	7
Pisum sativum	pea	seed			1	1		1								1			1				1	
Vicia ervilia	bittervetch	seed			8	2	3	2	2				1	2				3	1	4	3	4	25	14
Fabaceae (indeterminate)	legumes	seed			1						1		1											
**Wild Plants**																								
Allysum sp.	sweet alyssum	seed					1								1									
Arnebia sp.	arnebia	seed																						
Apiaceae	parsley family	seed							1		1													3
Brassicaceae	mustard family	seed			3		1							11	8	274		7		5	2	17	2	11
Bromus sp.	bromes	grain				22	5	1	31	1	4		18		2									
Bupleurum sp.	thoroughwax	seed																						1
Boraginaceae	borage family	seed												1	2	1			1			3		2
Carex sp.	sedges	seed					1	5		2											1			
Caryophyllaceae	pink family	seed				1									2						1			
Centaurea sp.	knapweeds	seed																						
Chenopodiaceae	goosefoot family	seed											2			1	1							
Chenopodium cf. *album*	baconweed	seed					2	3										1						
Capparis sp.	capers	seed													1	8		1	2		5	64	2	18
Cyperaceae	sedge family	seed			3	1				2	3	1	1		1	4					1	21		
Eleocharis sp.	needle spikerush	seed						1						1	3	41	1	1	7	3	8	4	4	8
Galium sp.	bedstraw	seed		1	5	5		5	13	4			7	2	2	23				2	1	11	1	
Hordeum spontaneum	wild barley	seed	1		2	2			4					2	6	39	1	6	3	1	1	5	1	12
Lithospermum arvense	field gromwell	seed	1	1	5	4	10	1	8	3	2	1	6											
Linum cf. bienne	pale flax	seed					1								1				1		3			
Malvaceae	mallow	seed				1	2	2													6			
Papaver cf. glaucum	tulip poppy	seed														2	2					2		6
Poaceae (indeterminate)	grasses	grain		1			1	1	11					1	1			1				1		
Poaceae	grasses	stem fragment											2			1								
Polygonaceae	knotweed family	seed					4	7																
Rosaceae	rose family	seed	1		1											1				1	1			5
Rubus sp.	blackberry	seed					2			2					1									
Rumex sp.	corly dock	seed			4				2															
Solonaceae	nightshades	seed					1									6	1	2	1		2			7
Salsola sp.	saltwort	seed				1	1		1	1			4					1			30			
Silene sp.	catchfly	seed												1	2	1			1					1
Thymelaeaceae	mezerium family	seed	1				3	4							2									1
Vaccaria pyramidata	cowherb	seed			9																			
Vaccaria sp.	cowherb	seed				1			1					4		5		1	2	3	1	2	4	1
Indeterminate		seed			2	3	3	1	1	3	1		2											
Food remain		fragmented piece						1					1			1					1			
